# Untangling Genomes of Novel *Planctomycetal* and *Verrucomicrobial* Species from Monterey Bay Kelp Forest Metagenomes by Refined Binning

**DOI:** 10.3389/fmicb.2017.00472

**Published:** 2017-03-29

**Authors:** John Vollmers, Martinique Frentrup, Patrick Rast, Christian Jogler, Anne-Kristin Kaster

**Affiliations:** ^1^Leibniz Institute DSMZ—German Collection of Microorganisms and Cell CulturesBraunschweig, Germany; ^2^Department of Microbiology, Institute for Water and Wetland Research, Faculty of Science, Radboud UniversityNijmegen, Netherlands

**Keywords:** algal biofilms, syntrophic interactions, biofouling control, natural product producers, genome binning

## Abstract

The kelp forest of the Pacific temperate rocky marine coastline of Monterey Bay in California is a dominant habitat for large brown macro-algae in the order of *Laminariales*. It is probably one of the most species-rich, structurally complex and productive ecosystems in temperate waters and well-studied in terms of trophic ecology. However, still little is known about the microorganisms thriving in this habitat. A growing body of evidence suggests that bacteria associated with macro-algae represent a huge and largely untapped resource of natural products with chemical structures that have been optimized by evolution for biological and ecological purposes. Those microorganisms are most likely attracted by algae through secretion of specific carbohydrates and proteins that trigger them to attach to the algal surface and to form biofilms. The algae might then employ those bacteria as biofouling control, using their antimicrobial secondary metabolites to defeat other bacteria or eukaryotes. We here analyzed biofilm samples from the brown macro-algae *Macrocystis pyrifera* sampled in November 2014 in the kelp forest of Monterey Bay by a metagenomic shotgun and amplicon sequencing approach, focusing on Planctomycetes and *Verrucomicrobia* from the PVC superphylum. Although not very abundant, we were able to find novel *Planctomycetal* and *Verrucomicrobial* species by an innovative binning approach. All identified species harbor secondary metabolite related gene clusters, contributing to our hypothesis that through inter-species interaction, microorganisms might have a substantial effect on kelp forest wellbeing and/or disease-development.

## Introduction

Submarine kelp forests are one of the most species-rich, structurally complex, and productive ecosystems in temperate waters. They are extremely well studied in terms of trophic ecology (Dayton and Tegner, 1984; Steneck et al., 2002; Estes et al., 2004; Graham, 2004) and provide habitat and nutrition to diverse communities extending from microorganisms to mammals. Kelp forests are highly diverse at the phylum level (Steneck et al., 2002; Graham, 2004) and—like tropical rain forests—important habitats and hot spots of biological diversity. Given that individual kelp algae can grow up to 50 cm in length every day, kelp forests are also mayor players in CO_2_ fixation in temperate waters (Foster et al., 2012). The so-called giant kelp, *Macrocystis pyrifera*, dominates these ecosystems along the temperate west coasts of North America (Dayton, 1985; Foster and Schiel, 1985; Delille and Perret, 1991; Graham et al., 2007). Along the central coast of California the kelp forest is of tremendous importance for coastal biodiversity, productivity, and the human economy and kelp-surface-associated bacteria are believed to be important players in carbon and nitrogen turnover in this food web (Linley and Field, 1982; Graham, 2004). While this habitat has been intensively researched for decades, still little is known about the microorganisms associated and interacting with the kelp, as only recently molecular techniques have become available to study biofilm species composition and abundance (Bengtsson et al., 2010, 2011, 2012; Hollants et al., 2013; Michelou et al., 2013).

Bacteria-algae interactions include symbiotic and parasitic relationships and mainly depend on environmental parameters, such as the availability of inorganic nutrients and organic matter. In eutrophic coastal marine systems like the Monterey Bay kelp forest rapid bacterial biofilm colonization takes place. Heterotrophic microorganisms are most likely attracted by the macro-algae through secretion of specific carbohydrates and proteins that trigger them to attach to their surface and to form biofilms, while degrading complex algal polysaccharides (Bengtsson et al., 2011, 2012; Jeske et al., 2013; Wegner et al., 2013). Kelp exudates may shape bacterial community composition, and create communities that are kelp-specific rather than randomly assembled from the surrounding seawater (Taylor et al., 2004; Longford et al., 2007; Reis et al., 2009). A study by Michelou et al. (2013) with samples taken from Monterey Bay in the months March and May in 2010 using 454-tag pyrosequencing of 16*S* rRNA genes showed that bacterial community structure and membership correlated with the kelp surface serving as host, and varied over time. *M. pyrifera* surface was enriched with *Rhodobacteraceae, Sphingomonadaceae (Alphaproteobacteria), Flavobacteraceae, Saprospiraceae (Bacteroidetes)* families and unclassified *Gammaproteobacteria*. Interestingly, sequences from the phyla *Verrucomicrobia* and Planctomycetes were detected, although not in high abundance. Several taxa were highly similar to other bacteria known to either prevent the colonization of eukaryotic larvae or exhibit antibacterial activities, which holds true for *Verrucomicrobia* and Planctomycetes (Wagner and Horn, 2006; Rao et al., 2007).

Studies of biofilms from the kelp algae *Laminaria hyperborea* collected along the west coast of Norway showed *Verrucomicrobia* and Planctomycetes to be even among the most frequently detected lineages (Bengtsson and Øvreås, 2010). However, biofilm composition was subject to seasonal variations (Bengtsson et al., 2010) and due to the dominance of few abundant Operational Taxonomic Units (OTUs), the kelp surface was characterized as low-diversity habitat (Bengtsson et al., 2012). Given the slow growth of *Planctomycetal* species (Fuerst, 2013) their abundance in such habitats, that are packed with carbon sources in contrast to the largely oligotrophic surrounding water, appears counter intuitive (Lage and Bondoso, 2014). Most other heterotrophs that dwell in such ecological niches divide much faster (for example 1.2–6.3 h for *Roseobacter* species (Christie-Oleza et al., 2012; Hahnke et al., 2013) and should generally outcompete slowly growing competitors. However, the interactions with the algae might involve the production of various secondary metabolites that are antimicrobial (defense against other, faster growing, heterotrophic bacteria) or algicidal (to destroy other eukaryotes like algae, diatoms or cyanobacteria for scavenging), and algae use those prokaryotic species as biofouling control (Zheng et al., 2005; Goecke et al., 2010). Those inter-species interactions of algae and bacteria and their resulting natural products are however not well understood (Estes et al., 2004).

**Figure 1:**
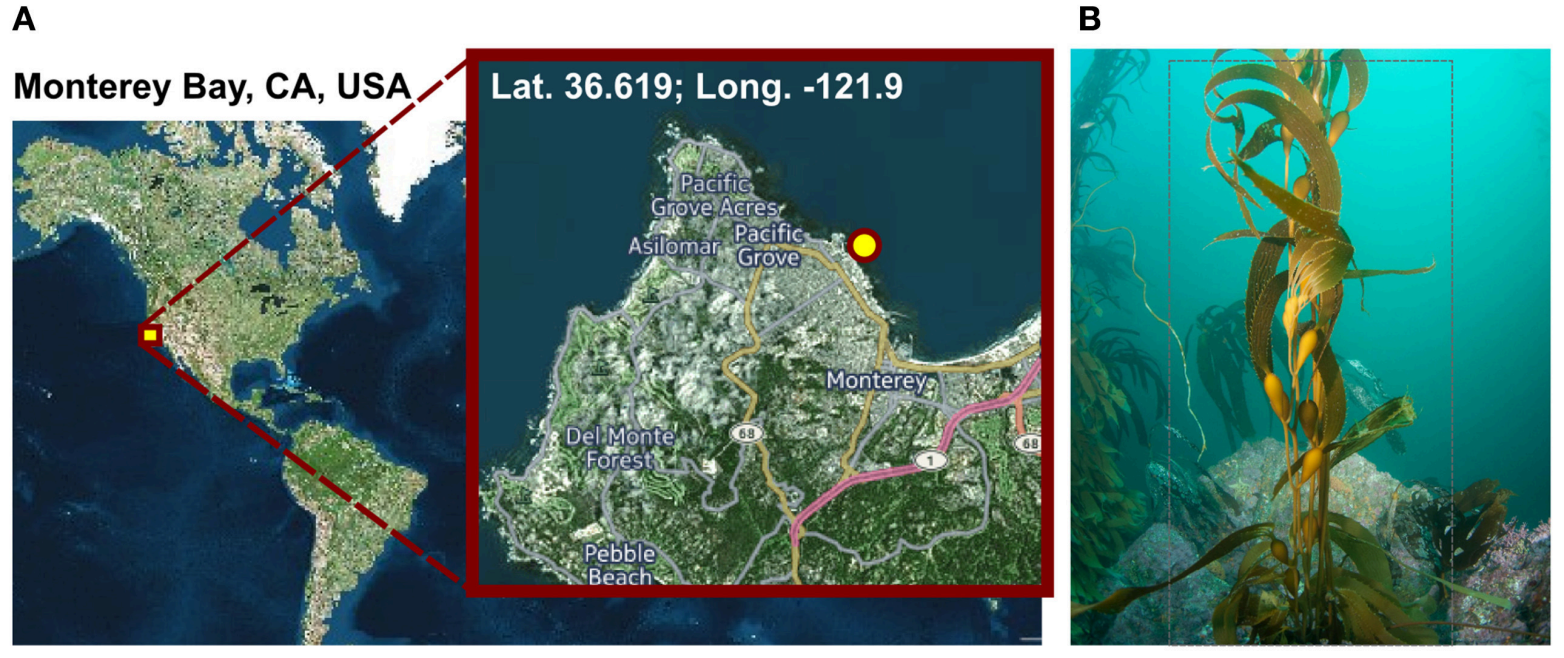
**Sampling. (A)** Geographic position of the sampling site. Visualization was done with cartoDB (https://www.carto.com). **(B)** *Macrocystis pyrifera* (kelp) specimen photographed at the sampling location during sampling in November 2014 at a water depth of 6 m and a water temperature of 12°C.

We here analyzed for the first time a biofilm sample from *M. pyrifera* of the Monterey Bay kelp forest by a metagenomic shotgun and amplicon sequencing approach with a focus on the PVC superphylum (Figure 1). This study reports an in-depth description of the diversity and phylogenetic association of the microbial communities associated with *M. pyrifera*. Through inter-species interactions Planctomycetes and *Verrucomicrobia* might have a substantial effect on kelp forest wellbeing or disease-development, providing a foundation for understanding the microbial ecology of kelp forests.

## Materials and methods

### Sampling

*Macrocystis pyrifera* was collected in 6 m water depth at a temperature of 12°C from the kelp forest near the Monterey Bay Aquarium, California, USA (lat. 36.619; long. −121.901) in November 2014 (Figure 1). Samples were stored in sterile Artificial Sea Water (ASW; 0.8 M NaCl, 0.06 M Na_2_SO_4_, 0.1 M MgCl_2_× 6 H_2_O, 19.5 mM CaCl_2_ × 2 H_2_O, 4.6 mM NaHCO_3_, 18.5 mM KCl, 1.6 mM KBr, 0.08 mM SrCl_2_ × 6 H_2_O and 0.14 mM NaF) and shipped on ice to Germany the same day. Upon arrival, the algae were cut into several 5 cm^2^ pieces and its biofilm was partially scraped off into 20 ml fresh ASW using a sterile scalpel, in order to achieve a partial enrichment of biofilm associated bacteria and to circumvent extracting eukaryotic cell material. However, since the biofilm was found to be thin and not always clearly visible, original kelp pieces were also retained for subsequent DNA extractions. Kelp pieces and scraped-off biofilm were stored separately in fresh ASW at −20°C until further processing.

### DNA extraction

In order to ensure a comprehensive representation of the kelp biofilm community, while minimizing eukaryotic DNA contamination from the algae itself and to enable a differential coverage binning approach, two different extraction methods were used to obtain DNA from kelp biofilm, resulting in DNA extracts A and B, respectively. Both extracts originated from the same kelp stipe, but from different blades. (**Extract A)** Sub-segments of one 5 cm^2^ kelp piece and 1.5 ml of scraped-off biofilm suspension were combined and subjected to pulse vortexing as well as 5 min of vigorous shaking in order to detach and capture tenacious biofilm community members which may not have been efficiently scraped off. Eukaryotic cells were then removed from the suspension via gravity flow filtration using a polycarbonate filter with 10 μm pore size (Celltrics filter, Partec, Münster, Germany). **(Extract B)** In order to minimize carry-over of eukaryotic cell fragments and to protect sensitive community members from shearing forces, 2 ml of undisturbed scraped-off biofilm suspension were carefully transferred to a new microcentrifuge tube without including the precipitate of residual algae fragments which formed by natural sedimentation. Subsequently, all DNA extraction steps were identical for both approaches, beginning with a two-step protocol adopted from Ferrera et al. (2010). Microbial cells were harvested by centrifugation (40 min, 16,000 g, 4°C) and re-suspended in 950 μl lysis buffer (40 mM EDTA, 50 mM Tris-HCl, 0.75 M sucrose). The cell suspensions were enzymatically lysed under slight shaking for 45 min at 37°C using 1 mg/ml lysozyme (final conc.), followed by chemical lysis under slight shaking for 60 min at 55°C using 1 mg/ml SDS and 0.2 mg/ml proteinase K (final conc., respectively). Two rounds of phenol:chloroform:isopentanol [25:24:1; v:v:v] extractions and two rounds of chloroform:isopentanol [24:1; v:v] extractions were performed. DNA of each sample was precipitated for 12 h at −20°C, using 0.3 M sodium acetate (final conc.) and 1 volume of ice-cold isopropanol, washed twice with 70% ethanol and re-suspended in 20 μl of nuclease free water. DNA purity was verified photometrically using a Nanodrop 2,000 Spectrometer (Thermo Fischer Scientific, Wilmington, USA), while DNA yield was determined using a Qubit® 1.0 Fluorometer with an dsDNA HS Assay Kit (Life Technologies, Darmstadt, Germany).

### Metagenomic shotgun library preparation

Separate shotgun libraries, KelpA and KelpB, were produced for DNA extracts A and B, respectively. DNA was sheared using a Covaris® S220 sonication device (Covaris Inc; Massachusetts, USA; 55.5 μl shearing volume, 175 W Peak Incident Power, 5% Duty factor, 200 burst cycles, 55 s treatment time). Sequencing libraries were prepared using the NEBnext® Ultra™ DNA Library Preparation Kit for Illumina® (New England Biolabs, Frankfurt, Germany) as per the manufacturer's instructions. Due to a high concentration of inhibitory contaminants, such as algal osmolytes released during sample treatment, extract A had to be diluted prior to library preparation, limiting the respective amount of input DNA to 5 ng. Therefore, 12 cycles of enrichment PCR were performed during the preparation of library KelpA. Since extract B was free of such contaminants, the full DNA yield of 105 ng could be utilized for preparation of library KelpB, necessitating only 6 cycles of enrichment PCR.

### Amplicon library preparation

An aliquot of DNA extract A was diluted to 1 ng/μl with PCR-grade water and subjected to Whole Genome Amplification (WGA) using the illustra™ GenomiPhi™ V2 DNA amplification Kit (GE Healthcare) and a Veriti 96-Well thermal cycler (Applied Biosystems). In order to reduce stochastic amplification bias, 10 independent amplification reactions were performed, using 1 μl of diluted DNA in a 20 μl reaction volume each, and subsequently pooled. WGA products were quantified using a Qubit® 3.0 Fluorometer with the Qubit® dsDNA BR Assay Kit (Life Technologies, Darmstadt, Germany). The V3 region of the 16*S* rRNA gene was amplified from the pooled WGA products via two subsequent PCR steps. Both reactions were performed in 50 μl reaction volumes consisting of 0.02 U/μl Q5® High-Fidelity DNA Polymerase (New England Biolabs, Ipswich, MA, USA), 1 × Q5 high GC enhancer, 1 × Q5 reaction buffer and 200 μM dNTPs. While the applied temperatures varied, both PCR programs consisted of 10 amplification cycles, with 1 min each for denaturing, annealing and elongation, as well as a preliminary denaturing step and a final elongation step of 5 min each. Reactions were performed in triplicates, which were subsequently pooled in order to reduce stochastic amplification bias. The first pre-amplification step was performed using 0.1 μM each of the V3 region specific forward primer 341f (5'- CCT ACG GGW GGC WGC AG-3') and the universal reverse primer uni515r (5'- CCG CGG CTG CTG GCA C-3') modified from 341f and 518r by Muyzer et al. (1993), respectively. Three hundred nano grams of WGA product was used as template. Cycling temperatures were 94°C for denaturing, 63°C for annealing and 72°C for elongation. Illumina® sequencing adapters and barcodes were added during the second amplification step following the pooling and purification of the pre-amplification products. For this step, 0.2 μM each of the extended V3 region primers “V3 *fwd”* (5′- AAT GAT ACG GCG ACC ACC GAG ATC TAC ACT CTT TCC CTA CAC GCT CTT CCG ATC TCC TAC GGG WGG CWG CAG -3′) and “index 34 V3 *rev”* (5′- CAA GCA GAA GAC GGC ATA CGA GAT TAT TCG GTG ACT GGA GTT CAG ACG TGT GCT CTT CCG ATC TCC GCG GCT GCT GGC AC −3′) were used, both of which were modified from Bartram et al. (2011). Fifteen microliters of pre-amplification product was used as template. Denaturing, annealing and elongation temperatures were 98, 65, and 72°C, respectively. PCR products were separated by electrophoresis using 2% agarose gels in 1 × Tris-Acetate-EDTA (TAE) buffer. The gel was stained with SYBR® gold (Thermo Fisher) for 1 h and inspected under UV light. Amplicon bands (~300 bp sized fragments) were excised and extracted using a spin column based approach (NucleoSpin Gel and PCR Clean-up; Macherey-Nagel).

### Sequencing and read processing

All libraries were sequenced on an Illumina® MiSeq using v3 chemistry, 301 cycles per read and paired end settings. Raw sequences were subjected to adapter clipping and quality trimming using Trimmomatic v.0.36 (Bolger et al., 2014) with the following arguments: “LEADING:3 TRAILING:3 SLIDINGWINDOW:4:15 MINLEN:105.” For preliminary metagenome assemblies, only the resulting read pairs were used, excluding orphans. For subsequent mapping and reassembly steps, as well as amplicon analyses, overlapping read pairs were identified and merged using FLASH v.1.2.11 (Magoč and Salzberg, 2011). Due to the short length of the amplicon inserts, amplicon read pairs overlapped in their entire sequence length. Therefore, merging resulted in high confidence consensus sequences which minimized the influence of random sequencing errors. Merged amplicon reads were furthermore analyzed and filtered based on the presence of the employed V3 region specific forward and reverse primer sequences, which were subsequently clipped from the reads.

### Metagenome shotgun assembly

Processed reads were pooled into a combined dataset KelpAB and assembled using IDBA-UD v.1.1.1 (Peng et al., 2012). For reference, additional individual assemblies were performed for each dataset, separately. In order to take optimal advantage of the longer MiSeq sequencing read lengths for correctly resolving repetitive regions, the source code was slightly altered to allow k-mer lengths of up to 251 bp, according to guidelines provided by the developers (https://github.com/loneknightpy/idba). For the preliminary metagenome shotgun assemblies, only paired reads were used, as IDBA_UD is not optimized for unpaired or merged reads.

### Binning and reassembly

Initial binning was performed using Maxbin v.2.1.1 (Wu et al., 2016) yielding 136 bins (Supplementary Table 1). After analysis and classification of these preliminary bins using CheckM v.1.0.4 (Parks et al., 2015), 8 *Planctomycetal* bins and 2 *Verrucomicrobial* bins were selected for further processing, in order to improve the assembly and remove residual contaminating genome fragments (Table 1). Putative contaminants were identified by taxonomically classifying the scaffolds of each bin using the Least Common Ancestor (LCA) approach implemented in MEGAN5 (Huson and Weber, 2013). Classifications were based on blastx and blastn comparisons against the NCBI nt and nr databases respectively, using an evalue cutoff of 1e-20. Only scaffolds that could be unambiguously assigned to any other phyla than the expected Planctomycetes or *Verrucomicrobia* were removed as putative contaminants. Subsequently, the average read coverage of each metagenomic scaffold was determined by mapping the sequencing datasets KelpA and KelpB individually against the combined assembly (KelpAB) using bowtie2 (Langmead and Salzberg, 2012). This information was used to analyze the coverage distribution within each of the selected bins. All outliers, consisting of scaffolds with coverage values larger than three-fold the median of the respective dataset and bin, were removed. Furthermore, bins were examined for sets of scaffolds displaying inverse relative coverage profiles when comparing KelpA and KelpB (e.g., displaying high abundance in KelpA but low abundance in KelpB or vice versa) and divided into appropriate sub-bins if applicable. In order to enable a targeted re-assembly, the corresponding sequencing reads were extracted from datasets KelpA and KelpB based on bowtie2 mappings for each of the hereby pre-filtered bins. To maximize sequence information and coverage, orphaned reads and merged read pairs were included in the analyses. To ensure high specificity, the “end-to-end” setting of bowtie2 was employed accepting only reads aligning in the entirety of their length. Furthermore, read pairs were only accepted if both reads mapped to the respective bin. The resulting read subsets were individually reassembled using SPAdes v.3.7.1 (Bankevich et al., 2012) and utilizing the scaffolds of the respective purified preliminary bin as “untrusted reference.” For final purification, new coverage profiles were calculated based on bowtie2 mappings and analyzed in order to identify scaffolds displaying differential coverage between datasets KelpA and KelpB, indicating putative contaminating genome fragments in each bin. To this end, the coverage values for each bin and dataset were converted into z-scores representing the respective deviation from the mean coverage of each analyzed bin. Putative contaminants were identified as scaffolds displaying differences above a specified cutoff between the z-scores for datasets KelpA and KelpB. Z-scores were recalculated for the remaining scaffolds, each time a putatively contaminating scaffold was removed. This process was performed iteratively beginning at a z-score cutoff of 4, decreasing the cutoff by 1 each time no further contaminants could be identified, until a final cutoff value of 2 was reached (corresponding to a coverage difference of 2 x the standard deviation for each dataset). The final, processed bins were re-examined using CheckM with appropriate phylum specific marker sets for Planctomycetes or *Verrucomicrobia* (Table 1).

**Table 1:** **General statistics of Planctomycetes and ***Verrucomicrobia*** bins**.

**Bin**	**IMG/M analysis project ID**	**Completeness & Contamination**				**Assembly statistics**						**Relative abundance compared to total bacterial population for individual bins and grouped bins, respectively [KelpA/KelpB/KelpAB]**		
		**based on CheckM using phylum-specific markers**			**based on tRNAs**									
		**Compl. [%]**	**Cont. [%]**	**Strain heterogen. [%]**	**Compl. [%]**	**Genome size [bp]**	**No. contigs**	**N50 [bp]**	**Mean contig length [bp]**	**Longest contig (bp)**	**GC [%]**			
Planctomycetes bin 1	Ga0136817	84.11	2.28	44.44	>99	4.642.202	422	21.658	11.000	66.126	52.33	0.37%/0.6%/0.55% /0.55%		
Planctomycetes bin 2	Ga0136818	55.92	18.95	6.02	50	3.449.631	1.946	1.836	11.772.772	8.106	52.33	0.01%/0.05%/0.04%/ % / 0.04%		
Planctomycetes bin 3	Ga0136832	**48.51**	11.02	6.67	70	2.487.561	1.428	1.767	1.741	7.729	50.8	0.17%/0.1%/0.11%		
Planctomycetes bin 4	Ga0136833	**37.69**	7.88	0	15	2.331.893	1.507	1.503	1.547	8.668	53.1	0.06%/0.1%/0.09%	0.62%/ 0.86%/ 0.81%	
Planctomycetes bin 5	Ga0136834	**39.31**	8.4	17.14	55	2.181.529	1.181	1.942	1.847	7.171	52.3	0.02%/0.01%/0.01%		1.14%/ 1.14%/ 1.14%
Planctomycetes bin 6	Ga0136835	13.82	3.52	21.05	85	1.947.306	1.261	1.492	1.544	7.283	48.5	0.04%/0.11%/0.1%		
Planctomycetes bin 7	Ga0136836	9.23	0.8	0	10	1.142.748	715	1.539	1.598	9.254	48.3	0.1%/0.12%/0.12%		
Planctomycetes bin 8	Ga0136837	8.5	0	0	30	880.382	612	1.380	1.438	4.317	54	0.37%/0.04%/0.11%		
Verrucomicrobium bin 1	Ga0136838	**47.77**	0.41	0	45	1.639.567	806	2.209	2.034	9.554	47.8	0.84%/0%/0.17%	0.87%/0.09%/0.25%	
Verrucomicrobium bin 2	Ga0136839	**23.01**	0.41	0	20	643.037	438	1.433	1.468	4.804	47.2	0.03%/0.09%/0.08%		

**Figure 2:**
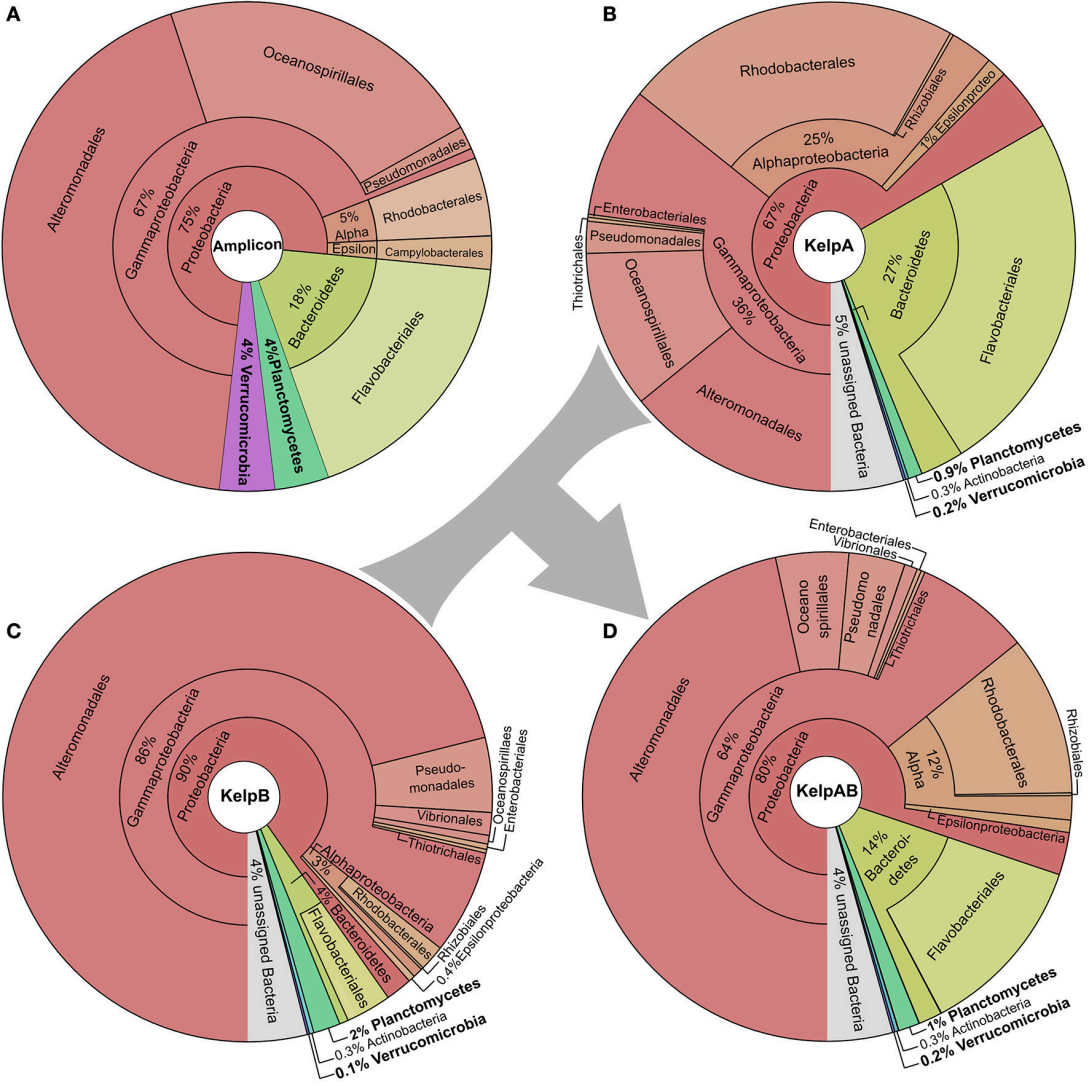
**Taxonomic composition of the kelp biofilm community**. Pie charts showing the relative abundances of sequences associated with bacterial taxa within each sequencing dataset down to order level. Visualizations were done using KRONA (Ondov et al., 2011). **(A)** 16*S* rRNA amplicon dataset. Relative abundances were determined using the SILVA NGS pipeline (Quast et al., 2013; Yilmaz et al., 2014). **(B,C)** Metagenomic shotgun libraries of KelpA & KelpB, respectively. KelpA **(B)** was derived from the same sample as the amplicon library, while KelpB **(C)** was derived from a separate sample obtained from a different leaf of the same Kelp specimen, using a slightly modified DNA extraction protocol. Relative abundances were determined based on sequencing read classifications obtained by using the Least Common Ancestor (LCA) approach implemented in MEGAN5 (Huson and Weber, 2013). In order to maximize coverage for low abundant community members, both datasets were treated as a composite dataset KelpAB **(D)** for assembly.

*Detailed completeness and contamination estimations were based on CheckM v.1.0.4 (Parks et al., 2015) results using phylum-specific marker sets. “Contamination” values indicate the percentage of phylum-specific marker genes, which were found in multiple copy number and do not necessarily reflect actual contamination. Similarly, “Strain heterogeneity” values indicate the percentage of multi-copy marker genes with more than 90% sequence identity and may not reflect actual heterogeneity. Bins were sorted and numbered, based on estimated completeness. Planctomycetes bins are marked in shades of green, Verrucomicrobium bins are marked in shades of violet. Bins with completeness values lower than 20% are marked in gray, while completeness values above 20% are marked by bold lettering. Additional rough estimations of genome completeness, based on distinct tRNA species, are included for reference. Relative abundances were based on the average read coverage of each bin after mapping the unassembled reads to the combined metagenome assembly. Combined abundance values for different combinations of planctomycetal and verrucomicrobial bins are shown in light green for Planctomycetes bins and in light violet for Verrucomicrobia bins. The relative abundances of *Planctomycetal* and *Verrucomicrobial* bins were found to be in accordance with estimations based on unassembled reads (Figure 2) as well as reconstructed 16S rRNA genes (Supplementary Table 5)*.

### Annotation and functional analyses

Gene calling and annotation was performed for processed bins using the Prokka pipeline v.1.12 (Seemann, 2014). Additionally, genes were annotated with SEED categories, based on blastp alignments against the NCBI nr database and subsequent LCA analyses using MEGAN5. AntiSMASH v.3.0 was employed to identify putative secondary metabolite gene clusters (Weber et al., 2015).

### Analyses of shotgun metagenomic 16*S* rRNA genes

RNAmmer v.1.2 was used to identify almost full-length (>1,100 bp) 16*S* rRNA gene sequences in the assembled metagenomes (Lagesen et al., 2007). However, due to the highly conserved nature of 16*S* rRNA genes, such sequences are likely to represent chimeras of different related strains when obtained by metagenome assembly. Therefore, the dedicated software tool EMIRGE (Miller et al., 2011) for reconstructing 16*S* rRNA genes from short read metagenomic data, was additionally employed for verification purposes. The resulting 16*S* rRNA sequences were aligned to the SILVA reference database and taxonomically classified using SINA (Pruesse et al., 2012; Quast et al., 2013; Yilmaz et al., 2014). Additionally, the sequences were phylogenetically clustered using a neighbor joining approach with 1,000 bootstrap iterations as implemented in ARB v.6.0.2 (Ludwig et al., 2004). A combination of phylogenetic, Multi Locus Sequence Analyses (MLSA) and coverage analyses was used to identify unambiguous connections between metagenomic bins and 16*S* rRNA gene sequences. Classification and relative abundances were visualized using the software tool Krona (Ondov et al., 2011).

### Analyses of 16*S* rRNA amplicon sequences

The low sequence length and high throughput of the amplicon sequencing data required a slightly different analyses pipeline than the almost full-length sequences reconstructed from the shotgun metagenome datasets. However, the same reference database as well as general taxonomic classification framework was used. Classification of processed amplicon reads was performed using the SILVAngs service platform and associated analysis pipeline (Quast et al., 2013; Yilmaz et al., 2014). Reads were uploaded as suggested in the SILVAngs user-guide and processed by the SILVAngs software according to the recommended protocol, including sequence alignment with the SINA aligner (Pruesse et al., 2012). During the process, a de-replication step, eliminating 100% identical reads, as well as OTU definition and clustering was performed. OTUs were classified by a local BLAST search using blastn with default parameters in accordance to the non-redundant version of the SILVA SSU Ref database. Classification and relative abundance of defined OTUs was visualized using the implemented software tool Krona (Ondov et al., 2011).

### Multi locus sequence analyses (MLSA)

Proteinortho5 (Lechner et al., 2011) was used to detect groups of orthologous genes shared between selected reference genomes and processed metagenomic bins. Reference genomes were obtained from NCBI and IMG/M. For each set of bins and comparison genomes, the respective “unique core genome” was determined as the list of gene products universally present in single copy. The size and composition of the resulting unique core genome set is strongly dependent on the reference genomes and the completeness of the analyzed bins. Therefore, separate analyses were performed for each processed metagenomic bin as well as combinations of bins associated with the same phylum. The resulting unique core genome gene products were individually aligned using MUSCLE v.3.8.31 (Edgar, 2004) and subsequently concatenated. Regions that could not be aligned were filtered from the alignments using Gblocks v.0.91b (Castresana, 2000). The filtered alignments were clustered using the neighbor joining algorithm with 1,000 bootstrap permutations, in order to reliably place each bin into a phylogenetic context.

### Accession numbers

Metagenome data and sequences of the 8 *Planctomycetal* and 2 *Verrucomicrobial* bins were deposited with the Integrated Microbial Genomes (IMG) database. KelpAB (coassembly): Ga0136809, KelpA (single assembly): Ga0136854, KelpB (single assembly): Ga0136855; *Planctomycetal* bin 1: Ga0136817, *Planctomycetal* bin 2: Ga0136818, *Planctomycetal* bin 3: Ga0136832, *Planctomycetal* bin 4: Ga0136833, *Planctomycetal* bin 5: Ga0136834, *Planctomycetal* bin 6: Ga0136835, *Planctomycetal* bin 7: Ga0136836, *Planctomycetal* bin 8: Ga0136837; *Verrucomicrobial* bin 1: Ga0136838, *Verrucomicrobial* bin 2: Ga0136839.

## Results and discussion

We here analyzed the biofilm community of *M. pyrifera* sampled from Monterey Bay kelp forest in November 2014 on multiple levels: Amplicon sequences, unassembled metagenomic shotgun reads, and assembled metagenome (Figure 2, Supplementary Figures 1, 2). Amplicon library sequencing yielded in 660.000 high quality reads in overlapping pairs, which could be merged into 330.000 consensus reads (Supplementary Figure 1A). Metagenomic shotgun sequencing yielded 36 mio raw reads per sequencing library. After quality trimming, 28 mio high quality reads were retained for library of KelpA and 34 mio reads for KelpB. In both cases, a large fraction of ~23 mio reads formed overlapping read pairs, which could be merged into longer consensus sequences prior to assembly (Supplementary Figure 1B). The amplicon library based approach indicated a bacterial biofilm community comprised predominantly of Proteobacteria and Bacteroidetes, with Planctomycetes and *Verrucomicrobia* representing lower but nonetheless substantial community fractions of 4% each (Figure 2A). This observation is in concurrence with a previous amplicon based analysis of *M. pyrifera* associated communities sampled during spring 2010. The overall trend of this community profile was furthermore confirmed by the Least Common Ancestor (LCA) taxonomic classification of a metagenomic shotgun sequencing dataset (KelpA) obtained from the same sample (Figure 2B), albeit with pronounced differences in the exact proportions of each bacterial fraction. Among the most noteworthy differences between the amplicon and the shotgun sequencing approach are the considerably lower fractions of Planctomycetes and *Verrucomicrobia* in the latter, comprising only 0.9 and 0.2% of the bacterial community, respectively. A cause for these discrepancies might be the bias introduced by the Phi29 based Whole Genome Amplification (Lasken, 2009) used in the preparation of the amplicon, but not the metagenomic shotgun library. However, since correct classification of short shotgun reads is highly dependent on the presence of closely related genome sequences within public databases, this divergence probably reflects the low representation of the PVC superphylum within currently sequenced genomes as well as the uniqueness of the Planctomycetes and *Verrucomicrobia* strains present in the here sampled habitat. The latter assumption is partially supported by the fact that relative abundances of the PVC superphylum diverge by a factor of 4.4–20% between amplicon and metagenomic shotgun data sets, while most other phyla and classes diverge only by a factor of 1.5–5%. The underrepresentation of PVC-classified reads, caused by the generally high variability of protein coding genes in combination with the low number of currently available PVC reference genomes also increases the likelihood of PVC-associated reads being classified as “unassigned bacteria.”

In the past, PVC community members have been often reported to be highly abundant and sometimes even dominant in kelp biofilm communities (Bengtsson and Øvreås, 2010; Michelou et al., 2013). However, the dominance of specific species or OTUs is often only temporary (Bengtsson et al., 2012; Michelou et al., 2013), e.g., in form of seasonal blooms. Metagenomics data are therefore likely not generally representative for the respective environment but only for the specific set of conditions during sampling. In addition, virus infections might change a microbial community within hours. Therefore, crucial members of communities may only be found in low abundance most of the time, in some cases even always (K-strategists). Such organisms may be much harder to isolate and analyze, but represent a huge hidden genomic potential, which still remains to be discovered. Unfortunately, whether trying to obtain isolates from environmental samples, or trying to reconstruct genomes from metagenomes (Albertsen et al., 2013; Sangwan et al., 2016) the focus is most often laid on the most abundant community members. As a result, low abundant and/or slowly growing species are likely to be underrepresented in public databases. With our innovative binning approach we here provide insights into the genomes of some low abundance but ubiquitous *Planctomycetal* and *Verrucomicrobial* species on algae and were able to generate a good draft genome of a novel Planctomycete which cannot be assigned to any of the known genera with cultured representatives.

Since binning efficiency is significantly improved by employing multiple related datasets, two metagenomic shotgun libraries were produced using slightly altered extraction protocols and different blade sections of the same kelp specimen, resulting in the datasets KelpA and KelpB. Similar to KelpA, KelpB was dominated by Proteobacteria, particularly Gammaproteobacteria, and contained only relatively small fractions of Planctomycetes and *Verrucomicrobia* (Figure 2C). However, both datasets differed in the exact proportions of the individual observed taxa, caused by a combination of community variability within kelp specimens as well as differences in extraction protocols. Differences include a lower relative abundance of Planctomycetes in KelpA compared to KelpB, while the opposite seems to be true for *Verrucomicrobia*, indicating that coverage differences between these datasets may be utilized to improve subsequent binning steps. The most pronounced differences were observed for the class Alphaproteobacteria and the phylum Bacteroidetes, which are present in considerably lower abundance in KelpB compared to KelpA by factors of ~8 and ~7, respectively. This indicates that intra-host variation and differences in extraction methods may greatly influence the community structure observed by metagenomic analyses, but can be utilized for differential coverage binning approaches. While both libraries confirm the dominance of Proteobacteria observed in the amplicon library, the observed fraction of Planctomycetes and *Verrucomicrobia* is considerably lower, representing only 0.1–2% of the bacterial community. In order to maximize sequencing coverage for low abundant community members present in both sequencing datasets, KelpA and KelpB were pooled into the combined dataset KelpAB (Figure 2D) and re-assembled, prior to binning.

Unfortunately, the preliminary bins obtained using Maxbin v.2.0 were of mixed quality, with a large degree of fragmentation as well as contamination in most bins (Supplementary Table 1). In order to obtain the most reliable and representative portrayal of contained PVC superphylum members, selected bins were rigorously filtered and processed using a stringent custom pipeline (for details please see Materials and Methods). This resulted in one almost complete and seven partial *Planctomycetal* and two partial *Verrucomicrobial* bins (Table 1). However, the final processed bins did not include 16*S* rRNA sequences, since those were predominantly encoded on small genome fragments, which did not include any additional markers. Due to the conserved nature of 16*S* rRNA genes, such fragments could therefore not be unambiguously binned based on sequence composition.

**Figure 3:**
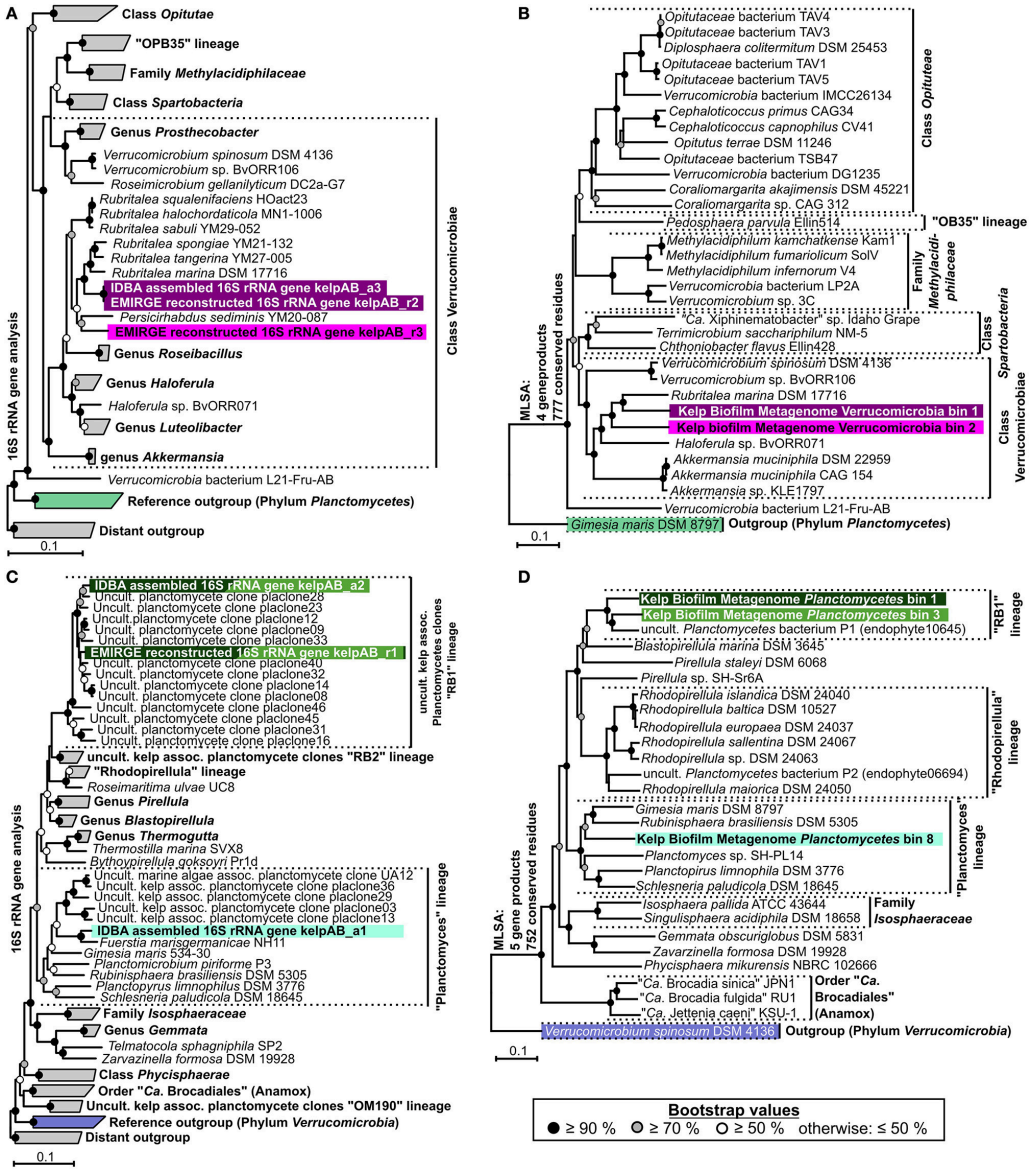
**Phylogenetic analyses. (A,C)** Neighbor joining trees showing the phylogenetic placement of ribosomal genes of *Verrucomicrobia* and *Planctomycetes*, respectively. Confidence values are based on 1,000 bootstrap permutations and indicated by appropriate symbols at each node. Ribosomal sequences and metagenomic bins obtained in this study are highlighted by different background colors representing putative different bacterial species & strains. Trees are based on aligned 16*S* rRNA genes >1,100 bp. For better presentability, outgroups, and several reference clades are shown in collapsed form. **(B,D)** Multi Locus Sequence Analyses (MLSA) based on unique core-genome gene products of *Verrucomicrobial* and *Planctomycetal* bins, respectively. The number of unique universally shared orthologs as well as the respective number of conserved amino acid positions considered for each analysis is indicated at the left of each tree. The exact unique core genome used for MLSA of different combinations of comparison genomes can be deduced from the results of the ortholog detection given in Supplementary Tables 3, 4. Due to the fragmented and incomplete nature of most metagenomic bins, relatively few universal orthologs could be considered in order to allow the simultaneous depiction of multiple representative bins. Higher phylogenetic resolutions can be found in the individual MLSA based phylogenies determined for each bin given in Supplementary Figure 3, which support the topologies depicted here. The MLSA results are in accordance with the observed 16*S* rRNA based phylogenies. Even though, due to strict binning parameters, none of the 16*S* rRNA genes depicted in **(A,C)** were included in any of the processed bins, each bin can be associated to a 16*S* rRNA gene based on phylogenetic placement as well as differential coverage information (Supplementary Table 5), as indicated by the respective background coloring (pink and purple = *Verrucomicrobia*, shades of green = Planctomycetes). Reference outgroups are marked in light violet (*Verrucomicrobia*) and light green (*Planctomycetes*)

Instead, two complementary methods were employed to obtain 16*S* rRNA sequences from the original metagenomic datasets: Direct extraction of 16*S* rRNA gene sequences using the hidden Markov model approach of RNAmmer, and the mapping based reconstruction approach implemented by EMIRGE. However, since the resulting genes do not represent clonal sequences they should be regarded as consensus sequences, analog to so-called Operational Taxonomic Units (OTUs). After checking for potential chimeras using DECIPHER (Wright et al., 2012), taxonomic classification using the LCA-approach of SINA, and screening for PVC-associated genes, at least two distinct sequences, representing separate organisms, were identified for each, *Verrucomicrobia* and Planctomycetes. Neither of those sequences could be assigned to a currently cultivated species (Figures 3A,C) and were therefore considered novel. An overview of the total extracted and reconstructed 16*S* rRNA sequences, as well as their taxonomic annotation and relative abundances, is given in Supplementary Table 2.

In order to enable a similar phylogenetic placement of the processed metagenomic bins, a Multi Locus Sequence Analysis (MLSA) approach was employed (Figures 3B,D, Supplementary Figure 3). The unique core genome used for MLSA of different combinations of genomes can be deduced from the results of the ortholog detection given in Supplementary Tables 3, 4. Since the MLSA- and 16*S* rRNA based phylogenies were fully coherent with each other, the resulting phylogenetic information was used in combination with the respective abundance profiles to link 16*S* rRNA sequences with the most likely corresponding bins (Supplementary Table 5). The two obtained partial *Verrucomicrobia* bins, cluster within the *Verrucomicrobiales*, with bin 1 being more or less directly associated with the family *Rubritaleaceae* (closest sequenced relative: *Rubritalea marina* DSM 17716 with an MLSA identity of 79%), and bin 2 being positioned between the *Rubritaleaceae* and the *Verrucomicrobiaceae* (closest sequenced relatives: *Rubritalea marina* DSM 17716 and *Haloferula* sp. BvORR071 with MLSA identities of 76 and 69%, respectively; Figure 3B). A highly similar topology is shown for the respective 16*S* phylogenies (Figure 3A), with the identical sequences KelpAB_r2 and KelpAB_a3 corresponding to bin 1 (closest relative: *Rubritalea marina* DSM 17716 with 95% identity) and KelpAB_r3 corresponding to bin 2 (closest relative *Persicirhabdus sedimis* YM20-087 with 93% identity, situated between *Rubritaleaceae* and *Verrucomicrobiaceae*).

The *Planctomycetal* bins 1–7 show on MLSA level the closest similarity to an uncultured Planctomycete genome P1 (MLSA identity values ranging from 73 to 79%), which was reconstructed from a red algae endophyte metagenome and is not to be confused with the *Rhodopirellula* sp. P1 (Bengtsson and Øvreås, 2010). Despite their relation, the observed phylogenetic distances imply that the here presented bins and the published uncultured endophyte Planctomycete genome P1 represent different taxa, at least on species and likely on genus level. This is supported by Average Nucleotide Identity (ANI) values of <90%, as determined using the Pyani package (https://github.com/widdowquinn/pyani), a value well below the commonly used species thresholds. Interestingly, the uncultured endophyte genome P1 contains a 195 bp long 16*S* rRNA gene fragment. While this fragment is unfortunately too short to be included in a comprehensive 16*S* rRNA based phylogenetic tree, BLAST comparisons revealed ~95% sequence identity to the kelp biofilm sequences KelpAB_r1 and KelpAB_a2, which are directly associated with the uncultured kelp associated “RB1” lineage. Therefore, and because of the similar tree topologies of MLSA and 16*S* rRNA based approaches (Figures 3 C,D), the aforementioned bins and the endophyte genome P1 could be unambiguously assigned to the “RB1” lineage, which has not previously been associated with genomic sequences to this day. This interesting lineage was previously shown to be the most abundant *Planctomycetal* group within biofilm communities on the surface of the kelp *Laminaria hyperborea*. Its members are highly diverse as indicated by the large number of closely related but distinct 16*S* rRNA gene sequences found in the respective clone libraries (Figure 3C). Assuming a similar presence of multiple closely related strains of the “RB1” lineage within the biofilm community of *M. pyrifera* would explain the high number and fragmented nature of the respective obtained *Planctomycetal* bins. Furthermore, this would explain the divergence between the “RB1” associated 16*S* rRNA genes obtained by the mapping based reconstruction approach (KelpAB_r1) and by direct assembly (KelpAB_a2). Such a divergence was not observed for *Verrucomicrobia*, where the 16*S* rRNA gene sequence obtained by direct assembly (KelpAB_a3) was identical to its respective counterpart obtained by the mapping based approach (KelpAB_r2). As a consequence, all attempts to reconstruct further genomes of the “RB1” lineage or to obtain respective isolates should take potential strain heterogeneity into account and justifying the strict bin processing approach employed in this study (please see Materials and Methods).

To this day, no genomic sequence fragments or isolate cultures have been directly associated with the “RB1” lineage. The here presented *Planctomycetal* bins 1–7 represent multiple closely related strains of this lineage in various degrees of fragmentation and completeness (Table 1), providing an interesting glimpse into the “RB1” pan-genome. Of these bins, *Planctomycetal* bin 1 possessed the highest quality, with a presumed completeness of up to 84.11% or even >99% based on marker gene and tRNA counts, respectively. The degree of putative contamination, determined using standard checkM quality analyses workflows with Planctomycetes specific marker sets was ~2%. This is far below the average value for other published *Planctomycetal* bins, since equivalent analyses of Planctomycetes genomes frequently yield “contamination” values of more than 3% and up to 7% (Supplementary Table 6). That is why this bin can be considered “pure” to the greatest possible extent. A linear visualization of *Planctomycetal* bin 1 shows that the contained scaffolds possess orthologs to reference isolate Planctomycetes throughout the binned genome (Supplementary Figure 4), with the most orthologs found in the uncultured endophyte reference genome P1, confirming a low, if any, degree of contamination. Nonetheless, distinct genomic islands are also recognizable as short stretches with relatively few orthologs in reference genomes, illustrating the large potential for new genomic features of this lineage.

The presence of a “RB1” associated OTU was also confirmed by a similar, but different, sequence obtained by the mapping based 16*S* rRNA reconstruction approach (kelpAB_r1), showing 97% identity to KelpAB_a2 and 98% identity to RB1 associated clone “placlone40.” Members of the “RB1”-lineage have been previously shown to be abundant and highly diverse within plasmid clone libraries of kelp-associated communities (Bengtsson and Øvreås, 2010). This diversity may be reflected in the observed distinct clustering of the two “RB1”-associated 16*S* rRNA gene sequences (kelpAB_a2 & kelpAB_r1) obtained in this study using separate methods, indicating that they may actually be consensus sequences representing multiple diverse but closely related strains. Interestingly sequence kelpAB_a1 clusters more closely to *Fuerstia marisgermanicae* NH11 (96% identity), a recently discovered novel *Planctomycetal* species, than to related plasmid clones obtained from other kelp-associated communities (Kohn et al., 2016).

In addition to the “RB1” associated bins, the low abundant and highly fragmented bin 8 as well as the corresponding 16*S* rRNA sequence KelpAB_a1, could be associated with the “planctomyces lineage.” This lineage contains a deep branching sub-group which clusters relatively close to, but nonetheless distinctly apart from several planctomycetes strains such as *Gimesia maris*. Interestingly, this deep-branching sub-group was also observed by Bengtsson and Øvreås (2010) to co-occur with the “RB1” lineage within kelp biofilm communities of *L. hyperborea* in relatively low abundance. However, the insights into the genomic potential of this sub-group are limited by the small size and low completeness of Planctomycete bin 8 (Table 1). Fortunately, the recently isolated strain *Fuersteria marisgermanica* (Kohn et al., 2016) proved to be not only associated with the aforementioned sub-group, but also most closely related to the here presented bin 8. Genome analyses of this species may provide more detailed insights into the potential role of this sub-group within marine biofilm communities.

All plancomycetal bins, with the exception of bin 8, contained genes encoding for flagellum synthesis. No such genes were identified in the *Verrucomicrobia* bins, which is in concordance with the non-motile lifestyle of their most closely related cultured representatives *Rubritalea marina* and *Persicirhabdus sediminis* (Scheuermayer et al., 2006; Yoon et al., 2008). Additional specifically noteworthy features identified in the PVC bins are listed in Supplementary Table 7.

**Figure 4:**
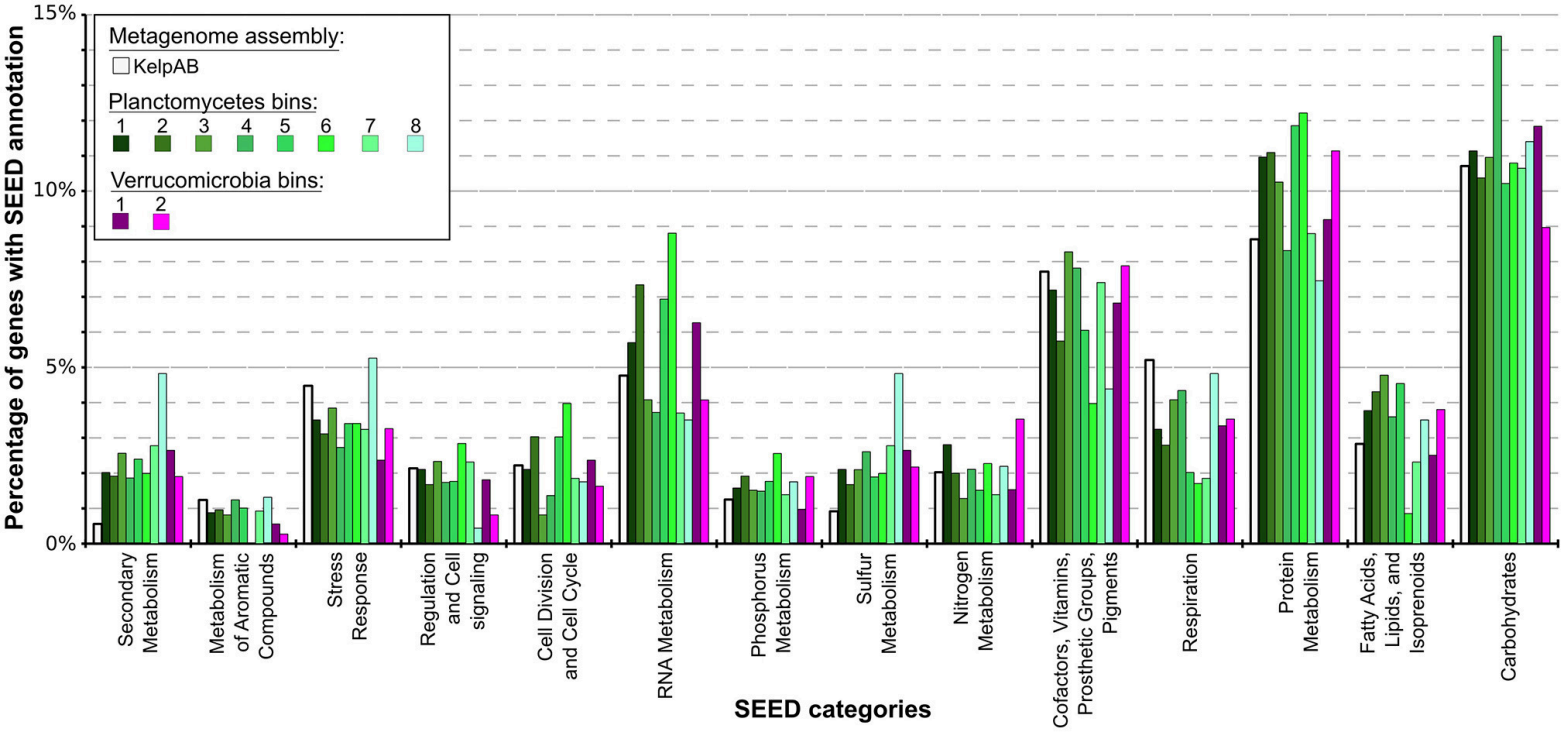
**Distribution of SEED category annotations for the metagenomic assemblies as well as each bin**. Relative fractions of selected SEED categories compared to the total number of SEED annotations in each assembly or bin are shown. ORF finding and gene prediction was performed using Prodigal (Hyatt et al., 2010). SEED annotations were obtained by aligning protein sequences against the NCBI nr database using Diamond (Buchfink et al., 2014) and analyzing the results using MEGAN5 (Huson and Weber, 2013). The *Planctomycetal* and *Verrucomicrobial* bins (shown in shades of green and violet, respectively) display two to four times higher proportions of gene products associated with secondary metabolism than average for the kelp biofilm community represented by the KelpAB metagenome (depicted in white), and a slightly higher fraction of genes associated with protein metabolism. Furthermore, genes associated with RNA metabolism are slightly more represented in *Planctomycetal* bins.

According to SEED annotations obtained via MEGAN5 analyses, a relatively high proportion of sulfur metabolism related genes was found in the *Planctomycetal* and *Verrucomicrobial* bins, which comprise a ~2–5 times higher fraction of the total SEED annotated genes compared to the total biofilm community (Figure 4; Supplementary Table 8). This observation is true for all of the processed PVC bins, including the almost-complete Planctomycetes bin 1, indicating that this is a general feature of the respective genomes and not biased by partial genome representation. The PVC-superphylum members might therefore represent the primary metabolizers of organic sulfur compounds within the kelp biofilm community. Such compounds are commonly produced in a large variety by marine macro algae (Sunda et al., 2002; Lage and Bondoso, 2014; Michel et al., 2006), and may therefore constitute key factors for the successful colonialization of, and adaptation to, algal surfaces. A high propensity for the utilization of sulfated glycopolymers has also been reported for several *Planctomycetes* and *Verrucomicrobia* cultures (Lage and Bondoso, 2011, 2012; Wegner et al., 2013; Spring et al., 2016). Furthermore, sulfated polysaccharides were the predominantly hydrolyzed glycopolymers during an unusual population spike of *Verrucomicrobia* observed at a Svalbard Fjord in 2008 (Cardman et al., 2014).

Other examples of nutrient scavenging which have been observed in different macroalgal associates include phosphorous and nitrogen utilization (Egan et al., 2013). Although a slight increase of genes encoding for these categories can be observed for several bins, including the almost complete Planctomycetes bin 1, this increase far less distinct compared to the aforementioned sulfur metabolism and not coherent for all PVC bins. The same holds true for genes involved in fatty acid, lipid and isoprenoid metabolism, which may be relevant for host-microbe interactions due to the production of polyunsaturated fatty acids (PUFAs) and terpenes frequently observed in marine macroalgae (Potin et al., 2002). Therefore, while it may be hypothesized that the PVC-superphylum members are also involved in the metabolisation of said nutrients and compounds in the kelp biofilm community, it is not conclusive whether they play a dominant role in this regard.

Genes encoding for respiration were found to be underrepresented in all of the PVC bins, indicating a relatively low metabolic activity. This might be contrasted by the comparatively high fraction of RNA metabolism related genes in several bins, which could be indicative for a complex regulatory network. These observations however do support the assumption that these genomes represent slow growing organisms, as frequently observed for PVC superphylum members.

**Figure 5:**
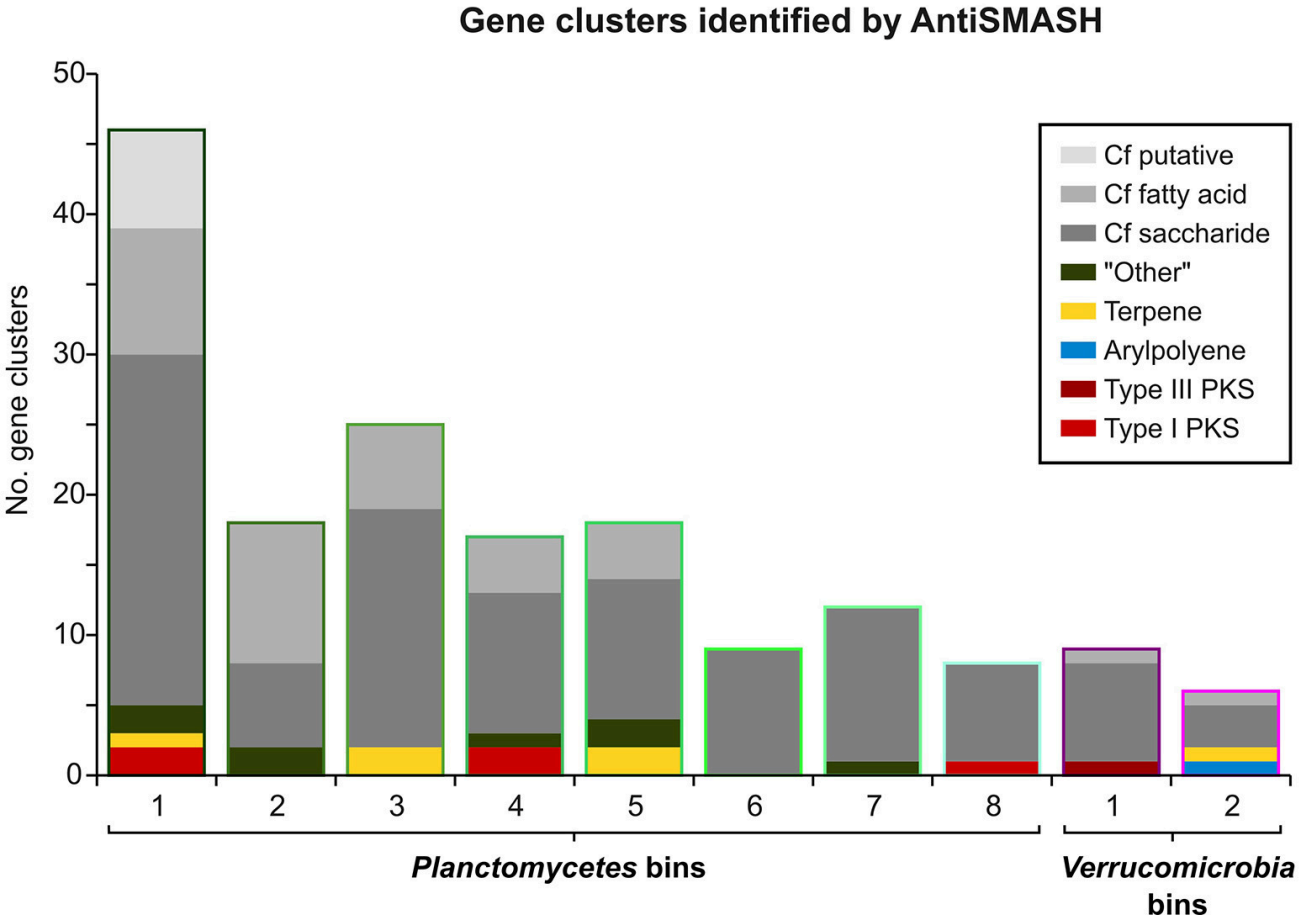
**Gene Clusters identified by AntiSMASH**. Bar heights indicate the total number of AntiSMASH hits identified in each bin. The respective relative fractions of different functional classes are indicated by distinct coloring.

Interestingly, an outstanding large relative fraction of the respective encoding genes in all of the processed bins are associated with secondary metabolism (Figure 4; Supplementary Table 8). This fraction is ~4–9 times higher in the *Planctomycetal* and *Verrucomicrobial* bins compared to the total kelp biofilm community and found evidence for a relatively high potential for secondary metabolite production in those species. Putative gene clusters were identified by AntiSMASH v.3 in all of the processed bins, including several potential Polyketide Synthase (PKS) clusters (Figure 5). PKSs are enzyme complexes or multi-domain enzymes which perform a stepwise biosynthesis of complex organic compounds, so-called polyketides, with varying pharmaceutical properties such as antibiotic activity (Jenke-Kodama et al., 2005). Nutrient rich habitats like kelp forests attract other –more rapidly- growing- heterotrophic bacteria that should be able to outcompete slow-growers. As *Verrucomicrobia* and Planctomycetes are both known to be relatively slowly growing organisms, secondary metabolites may likely serve as inhibitory agents against more rapidly growing competitors within the biofilm community. This would help explain the persistence and even frequent dominance of PVC superphylum members in kelp-associated communities, despite the constant danger of being out-competed by rapidly growing Proteobacteria. We therefore hypothesize that *Verrucomicrobia* and Planctomycetes are attracted by kelp through secretion of specific carbohydrates that trigger them to attach to the algal surface and to form biofilms. The kelp might then employ them as biofouling control, using their antimicrobial secondary metabolites to defeat other bacteria or eukaryotes. This would be in accordance with the low abundancy but ubiquitous distribution of these species on marine algae, and shows that these phyla are very interesting for natural products production/pharmaceuticals. Only recently, Planctomycetes were demonstrated to produce small molecules with bioactivity and their antibiotic effects were recently demonstrated (Jeske et al., 2013).

Although several studies have reported the discovery of new bioactive compounds from marine organisms and more than 15,000 structurally diverse bioactive compounds were isolated during the past 30 years (Hu et al., 2015), only two new classes of antibiotics have been brought to market during this period of time (Coates et al., 2011). In the post-genomic era one has come to realize that naturally occurring reservoirs of genetic diversity contain vast, untapped potential for production of biologically active chemical compounds that needs to be made available for society with the emerge of new technologies. The PVC superphylum is known for its biotechnological and medical relevance (Wagner and Horn, 2006) and a promising resource for natural products, however due to the relatively low representation of these phyla in public sequencing databases there are still may gaps to fill by e.g., specific isolation attempts or combinations of single cell (Rinke et al., 2013; Kaster et al., 2014) and metagenomics. Our study provides a first glimpse into this microbial dark matter by using an innovative binning approach to reconstruct genomes of low abundant species, shedding light on Planctomycetes and *Verrucomicrobia* from biofilms of kelp algae.

## Author contributions

AK and JV designed the experiments, performed the data analyses and wrote the paper. MF and PR performed the experiments. CJ collected the samples by scuba diving.

### Conflict of interest statement

The authors declare that the research was conducted in the absence of any commercial or financial relationships that could be construed as a potential conflict of interest.
