# Supplementary material for: Untangling Genomes of Novel Planctomycetal and Verrucomicrobial Species from Monterey Bay Kelp Forest Metagenomes by Refined Binning
Source: Front Microbiol. 2017 Mar 29;8:472. doi: 10.3389/fmicb.2017.00472 (PMC5372823; doi:10.3389/fmicb.2017.00472)
Supplement: Supplementary file 9 [file Image1.PDF]

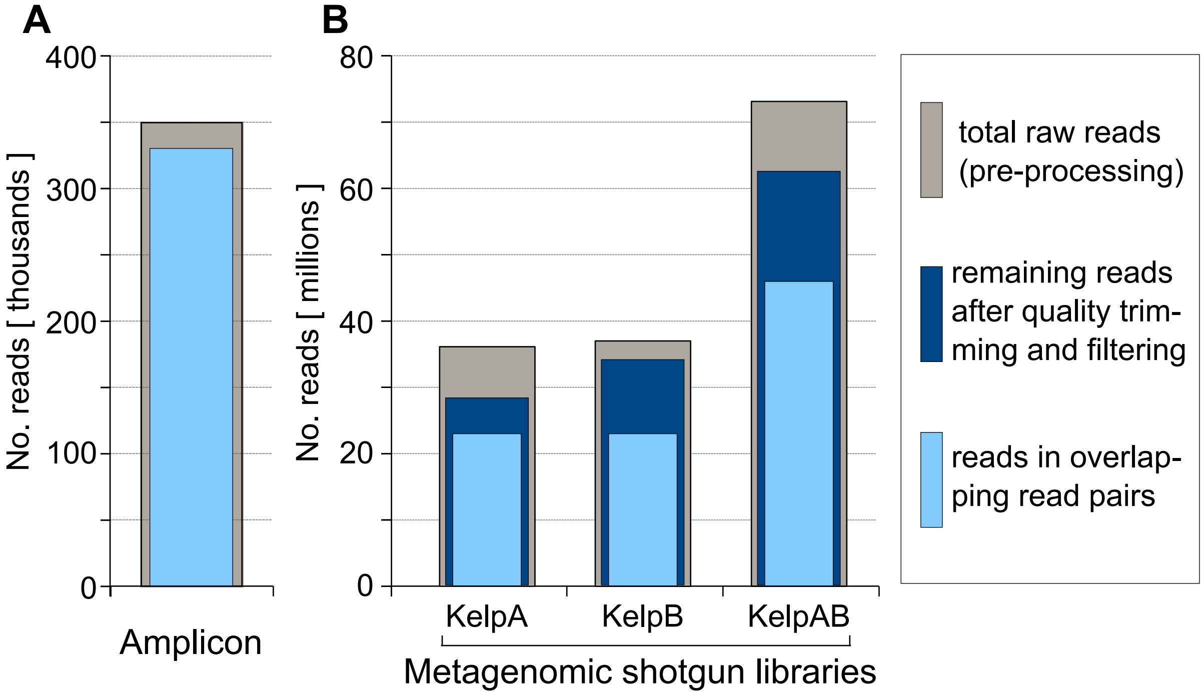

Supplementary Figure 1. Number of sequencing reads before and after processing. **A)** Number of sequencing reads for the amplicon library. **B.)** Number of sequencing reads for the single and combined metagenomic shotgun libraries, respectively. The respective fractions of total reads, quality trimmed reads and overlapping reads pairs are indicated by different colors. Overlapping read pairs were merged using FLASH (Magoč and Salzberg 2011) before assembly.
