# Supplementary material for: Untangling Genomes of Novel Planctomycetal and Verrucomicrobial Species from Monterey Bay Kelp Forest Metagenomes by Refined Binning
Source: Front Microbiol. 2017 Mar 29;8:472. doi: 10.3389/fmicb.2017.00472 (PMC5372823; doi:10.3389/fmicb.2017.00472)
Supplement: Supplementary file 10 [file Image2.PDF]

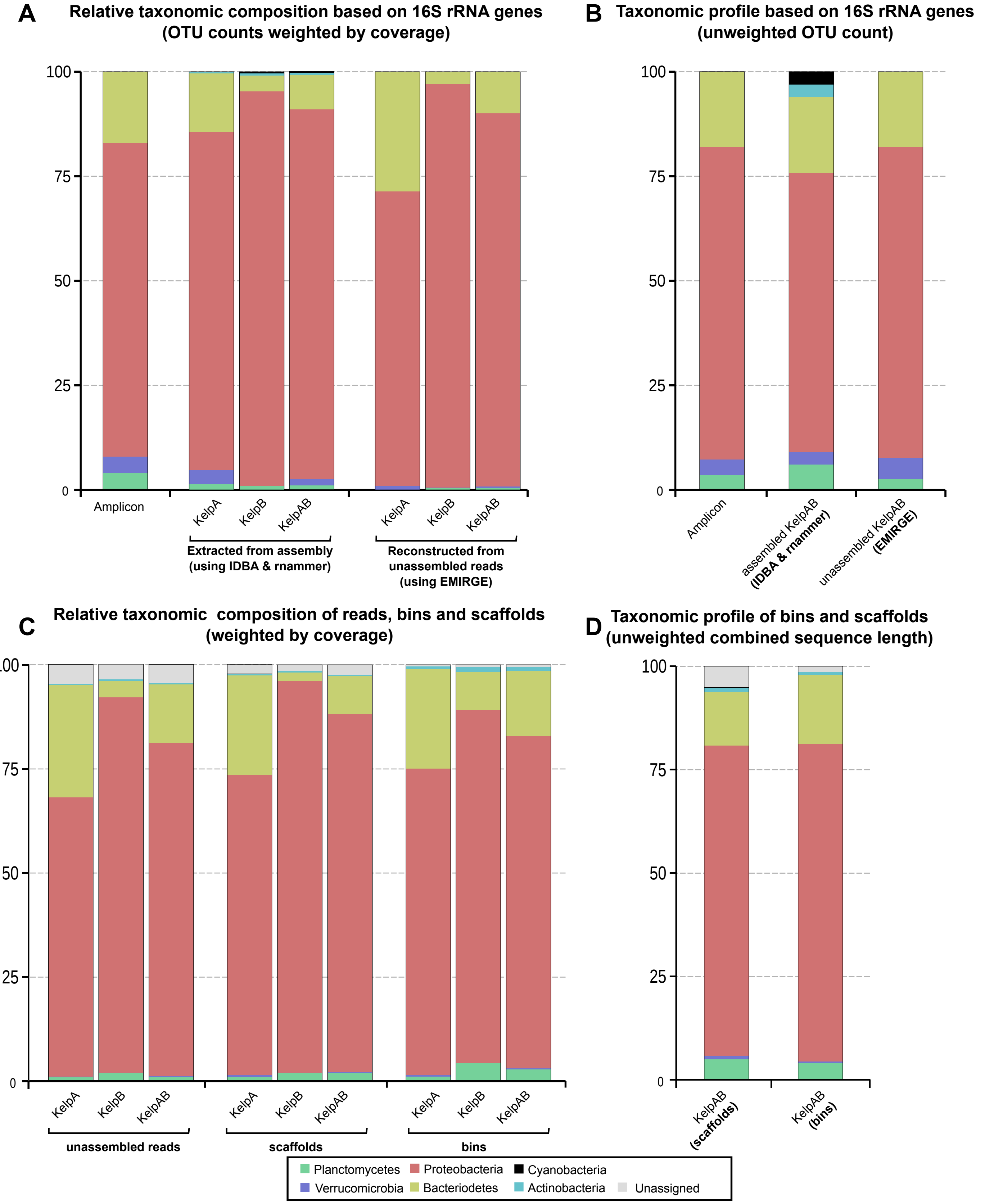

Supplementary Figure 2. Bar charts comparing the abundances of different phyla within the kelp biofilm datasets on 16S rRNA gene level **(A & B)**, and encoded protein sequence level **(C & D)**. **A)** Relative phylum composition based on relative OTU abundances calculated from associated read counts in the case of the amplicon library, coverage of the associated scaffold in the case of assembly derived 16S sequences, and normed prior probability values in the case of EMIRGE reconstructed 16S sequences (Miller, et al. 2011). Kelp A resembles the amplicon dataset more closely than KelpB and the combined Dataset KelpAB, which display lower relative abundances of Bacteroidetes, Verrucomicrobia and Planctomycetes. Furthermore, the assembly-derived 16S sequence dataset resembles the abundances within the amplicon dataset more closely than the read-derived 16S dataset obtained by using EMIRGE. B) Taxonomic profile based on the number of OTUs associated with each phylum, without considering relative abundances. The overall taxonomic profile is similar for all three types of 16S datasets. **C)** Relative phylum composition within the metagenomic shotgun datasets. Taxonomic classifications were based on blastx comparisons prior to assembly ('unassembled reads'), blastp comparisons after assembly ('scaffolds') and CheckM marker-gene analyses after binning ('bins'). Abundance values were calculated from associated read counts ('unassembled reads') or coverage values ('scaffolds' & 'bins'). The overall phylum composition remains highly similar at each level, indicating that no bias is introduced during the assembly and binning steps. However, since the confidence for sequences classification increases with the genomic context information at each step, the fraction of unassigned sequences and bins decreases. **D)** The relative proportion of assembled and binned sequence information associated with each phylum. Planctomycetes associated scaffolds and bins represent more than 5% of complete metagenome assembly. This is higher than expected from the relative abundance values and most likely caused by the relatively large size of Planctomycetes genomes
