# Supplementary material for: Untangling Genomes of Novel Planctomycetal and Verrucomicrobial Species from Monterey Bay Kelp Forest Metagenomes by Refined Binning
Source: Front Microbiol. 2017 Mar 29;8:472. doi: 10.3389/fmicb.2017.00472 (PMC5372823; doi:10.3389/fmicb.2017.00472)
Supplement: Supplementary file 11 [file Image3.PDF]

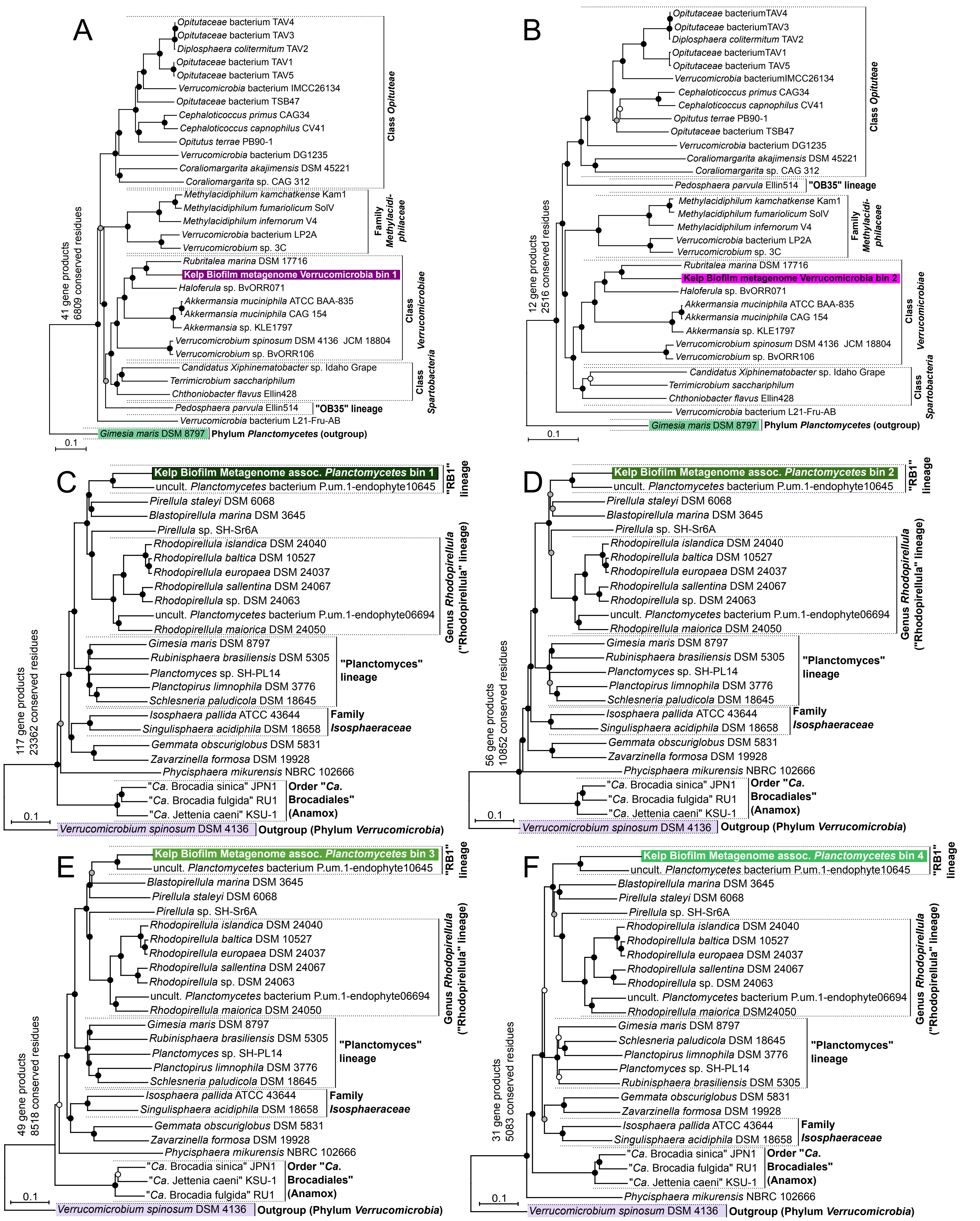

Supplementary Figure 3. Multi Locus Sequence Analyses (MLSA) based on unique core genome gene products of Verrucomicrobia and Planctomycetes bins, respectively. The number of unique universally shared orthologs as well as the respective number of conserved amino acid positions considered for each analysis is indicated at the left of each tree. The unique core genome used for MLSA of different combinations of comparison genomes can be deduced from the results of the ortholog detection given in Supplementary Tables 3 & 4.
