# Supplementary material for: Untangling Genomes of Novel Planctomycetal and Verrucomicrobial Species from Monterey Bay Kelp Forest Metagenomes by Refined Binning
Source: Front Microbiol. 2017 Mar 29;8:472. doi: 10.3389/fmicb.2017.00472 (PMC5372823; doi:10.3389/fmicb.2017.00472)
Supplement: Supplementary file 12 [file Image4.PDF]

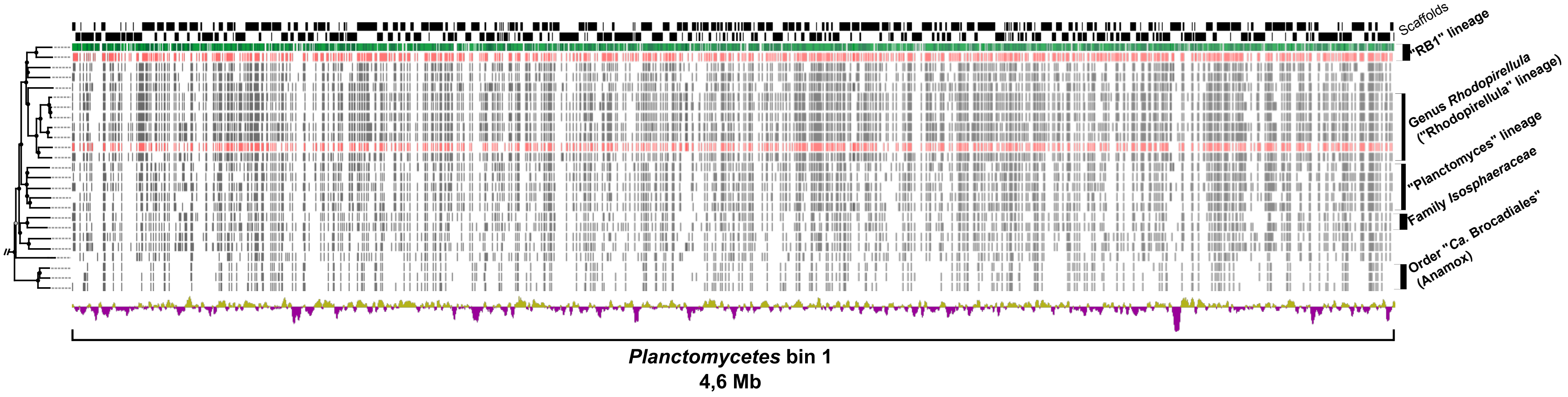

Supplementary Figure 4. Linear genome plot of Planctomycetes bin 1, showing the presence or absence of orthologs shared with reference genomes. Scaffolds are shown as black blocks in the first two lines. Scaffold order and orientation is random. Protein coding genes encoded in planctomycete bin1 are depicted in green in line three. The subsequent lines indicate the presence or absence of orthologs in each planctomycete reference genome. The order of reference genomes is the same as in the respective MLSA tree of **Supplementary Figure 3C**, which is also included on the left as reference. Reference genomes obtained from uncultured marine algae endophytes are highlighted in orange. The lower graph indicates the GC-content distribution, while the bottom scale indicates the overall genome size.
